# Clinical trial of carbon ion radiotherapy for gynecological melanoma

**DOI:** 10.1093/jrr/rrt120

**Published:** 2014-02-16

**Authors:** Kumiko Karasawa, Masaru Wakatsuki, Shingo Kato, Hiroki Kiyohara, Tadashi Kamada, the Working Group for Gynecological Tumors

**Affiliations:** 1 Research Center Hospital for Charged Particle Therapy, National Institute of Radiological Sciences, 4-9-1 Anagawa, Inage-ku, Chiba-city, Chiba 263-8555, Japan; 2 Department of Radiation Oncology, Saitama Medical University International Medical Center, 1397-1, Yamane, Hidaka-shi, Saitama, 350-1298, Japan; 3 Gunma University Heavy Ion Medical Center, 3-39-22 Showa-machi, Maebashi, Gunma, 371-8511, Japan

**Keywords:** carbon ion radiotherapy, gynecological melanoma

## Abstract

Carbon ion radiotherapy (C-ion RT) is an advanced modality for treating malignant melanoma. After we treated our first case of gynecological melanoma using C-ion RT in November 2004, we decided to conduct a clinical trial to evaluate its usefulness for the treatment of gynecological melanoma. The eligibility criteria for enrollment in this study were histologically proven malignant melanoma of the gynecological regions with lymph node metastasis remaining in the inguinal and pelvic regions. The small pelvic space, including the GTV and the metastatic lymph node, was irradiated with up to a total dose of 36 GyE followed by a GTV boost of up to a total dose of 57.6 GyE or 64 GyE in 16 fractions. A series of 23 patients were treated between November 2004 and October 2012. Patient age ranged from 51–80 with a median of 71. Of the tumor sites, 14 were located in the vagina, 6 in the vulva, and 3 in the cervix uteri. Of the 23 patients, 22 were irradiated with up to a total dose of 57.6 GyE, and 1 patient was irradiated with up to a total dose of 64 GyE. Chemotherapy and interferon-β were also used to treat 11 of the patients. Acute and late toxicities of Grade 3 or higher were observed in 1 patient treated with concurrent interferon-β. The median follow-up time was 17 months (range, 6–53 months). There was recurrence in 14 patients, and the 3-year local control and overall survival rates were 49.9% and 53.0%, respectively. C-ion RT may become a non-invasive treatment option for gynecological melanoma.

## INTRODUCTION

Primary melanoma of the gynecological organs is extremely rare, with the incidence of mucosal melanoma at 1.3% of all melanomas [1]. Vulval malignancies form 4% of gynecologic malignancies. Malignant melanomas (MMs) are ∼2–4% of primary vulval malignancies, and 1–3% of MMs arising in women [2–4]. Vaginal malignancies are 2% of gynecologic malignancies. MMs is about ∼ 3% of primary vaginal malignancies and <20% of genital MMs. MMs arising from the uterine cervix account for <1% of uterine malignancies [2–4].

For disease staging, various classifications including invasion depth of Breslow, Clark's level and Chung's level have all been tried. The UICC classification of skin melanomas has been reported to reflect a more accurate prognosis than the UICC classification of each gynecological malignancy [2–4]. Local control and survival rates are reportedly related to tumor thickness, ulceration, lymph node spread, and age [2–5].

Because of the low incidence of gynecological MM, it has as yet not been possible to establish an optimum treatment modality, and treatment results have mostly been reported as retrospective case report series. Surgical resection has to date been considered the best modality for this tumor. En-bloc excision with a safety margin is thought to be necessary for primary treatment, although not all patients are good candidates for surgery due to tumor extension, age, or physical condition. The median patient age for this tumor type is relatively higher than for other gynecological malignancies. Furthermore, even when total resection of the tumor has been performed, the outcome (in terms of local tumor control and long-term survival) has not been satisfactory, and postoperative physical and functional disabilities may be incurred [2–4].

Systemic therapy, such as dacarbazine monotherapy and DAVFeron (dacarbazine, nimustine, vincristine and interferon-β) therapy has been used in advanced or recurrent melanoma, and these days, molecular-target drugs such as vemurafenib and ipilimumab are being introduced. As yet, the results of chemotherapy treatment are insufficiently known, and efficacy of the molecular-target drug has not been established.

MM has been regarded as a radioresistant tumor, demonstrating poor regression after photon radiotherapy, even though the use of a high dose per fraction may improve local response up to a complete remission rate of 20–30% [1]. However, highly effective irradiation, such as high relative biological effectiveness (RBE) radiotherapy, may have the potential to become a definitive treatment for MM.

In 1994, C-ion RT was initiated at our institute, the National Institute of Radiological Sciences (NIRS) in Japan. Carbon ion beams provide superior physical dose distribution because of their finite range in the target tissue, and they possess a biological advantage due to their high RBE in the Bragg peak. It is therefore reasonable to assume that carbon ion beams might be superior to photons for managing tumors characterized by poor radiosensitivity, such as MMs [6]. At NIRS, we have achieved good local control of MM in the head and neck regions, as well as of choroidal melanoma [7, 8]. The 5-year local control rates achieved for advanced or unfavorably located choroidal melanoma and MM in the head and neck were reported as 92.8% and 75%, respectively [7, 8]. We treated the first case of gynecological melanoma using C-ion RT in November 2004, achieving good local control without severe adverse effects. After the first treatment, we decided to initiate a clinical trial for gynecological MM.

This article reports the retrospective analysis of C-ion RT in 23 patients with MM of gynecological regions.

## MATERIALS AND METHODS

### Eligibility

The eligibility criteria for enrollment in this study were (i) histologically proven MM of gynecological regions, (ii) localized measurable tumors, (iii) lymph node metastasis remaining in the inguinal lymph nodes and the pelvic region, with irradiation being possible in the same irradiation field, (iv) age between 20 and 80, (v) performance status 0–2, (vi) no critical complications or active double malignancy, and expectation of survival prognosis of at least 6 months, (vii) written informed consent.

The ineligibility criteria for enrollment in this study were (i) tumor extended to skin, (ii) less than 5 mm between the tumor and radiosensitive organs, including bowel and bladder, (iii) tumor with uncontrollable distant metastases, (iv) active intractable infection in an irradiation area, (v) prior radiotherapy in an irradiation area, (vi) patients determined to be unsuitable for treatment by doctor in charge.

All candidates were formally approved by the institutional review board before initiation of treatment.

### Study design and treatment

Between 2004 and 2012, all enrolled patients were irradiated by means of passive broad beam methods using individual collimators and a compensation bolus absorber. From 2012, the scanning beam method could also be applied [9]. At NIRS, a dose of carbon ions is expressed in photon equivalent doses (Gray equivalent dose: GyE), which is defined as the physical dose multiplied by the RBE of carbon ions. Biological flatness of the spread-out Bragg peak (SOBP) was normalized by the survival fraction of human salivary gland (HSG) tumor cells at the distal region of the SOBP where the RBE of carbon ions was assumed to be 3.0 [10, 11].

Patient evaluation included medical history, physical examination, magnetic resonance imaging (MRI) of the pelvis, computed tomography (CT) of the neck to pelvis, and 11C-methinine or 18F-fluorodeoxyglucose positron emission tomography (PET) was performed to distinguish clinical stage. Tumor thickness and the clinical T stage were decided by physical examination, CT and MRI. Before planning CT, an immobilization device was custom-made to hold the patient in the same position during the planning CT and radiotherapy. In 3D treatment planning, the gross tumor volume (GTV) was defined on the planning CT by fusion images with contrast enhanced CT, MRI and PET.

The clinical target volume (CTV)-1 included all areas of gross and potentially microscopic disease, consisting of the uterus, vagina and/or vulva, and the pelvic lymph nodes (internal iliac, external iliac, obturator) and inguinal lymph nodes, plus a 5-mm safety margin for positioning uncertainty. CTV-1 was irradiated with 36 GyE in 10 fractions via three portals, and was covered by at least 90% of the prescribed dose. CTV-2, defined as limited to the GTV and the GTV node with a minimum 5-mm margin, was then irradiated with up to a total dose of 57.6 GyE in 16 fractions via 2–3 portals. Organs at risk, such as the small intestine, colon, rectum and bladder, were excluded from the planning target volume (PTV) as much as possible. C-ion RT was given once daily for 4 days per week (Tuesday to Friday). For patient immobilization, a cast (Mold care; Alcare, Tokyo, Japan) and a fixation body shell (Shellfitter; Kerary Co., Ltd, Osaka, Japan) were made. To minimize organ motion, 100–150 ml of normal saline was infused into the bladder and the vagina was stuffed with cotton.

After April 2011, tumors of >60 ml were irradiated with up to a total dose of 64.0 GyE in 16 fractions referred to the treatment outcome of head and neck MM [12]. In these cases, the CTV1 was irradiated with 36 GyE in 9 fractions. The limiting dose for critical normal tissue was decided upon at 60 GyE for the bowel. The PTV was extended 3 mm from the CTV at the time of 290 MeV/n energy with the patient's individual collimator and 6 mm at the time of 350 MeV/n energy with the multi-leaf collimator.

Before and after the completion of C-ion RT, some patients received systemic therapy such as DAVFeron or Feron, depending on the decision of the attending gynecologist or dermatologist of each hospital. For patients with advanced disease and good general condition, we recommended the attending physicians do DAV-Feron therapy after completion of C-ion RT.

If local recurrence and/or distant metastasis were diagnosed, the treatment method for these tumors had no limitation and was decided upon by the attending gynecologist or dermatologist of each hospital.

### Evaluation

Patients were re-staged according to the seventh edition of the TNM staging system for each primary site (International Union Against Cancer; UICC, 2009). Furthermore, they were simultaneously classified according to the TNM of the skin melanoma, because there are some reports that classification of MM of the skin, including the degree of invasion, reflects the prognosis better than the classification of gynecological tumors according to tumor size [2–4]. Initial tumor response was determined as the maximum reaction within 6 months after the start of C-ion RT by physical examination, MRI, CT and PET.

Acute reactions of normal tissue were classified according to the Cancer Therapy Evaluation Program, Common Terminology Criteria for Adverse Events, Version 4.0 [13], with a maximum reaction within 3 months after initiation of therapy. Late reactions were classified according to the RTOG/European Organization for Research and Treatment of Cancer (EORTC) scoring system [14]. All patients were followed up with CT and MRI every 3 months for the first 2 years and every 6 months thereafter.

### Statistical analysis

The primary objectives of this study were to assess local control (LC), regional control (RC), distant control (DC), relapse-free (RF) and overall survival (OS). The secondary objectives were the assessment of acute and late normal tissue morbidities.

Probabilities of LC, RC, DC, RF and OS were calculated using the Kaplan–Meier method. Time-to-event was measured from the first day of C-ion RT. The log-rank test was used to evaluate the differences between the probability curves. All statistical analyses were performed using Stat Mate III (Atoms, Tokyo, Japan). A *P*-value <0.05 was considered statistically significant.

## RESULTS

### Patient characteristics

Between November 2004 and October 2012, 23 patients were treated with this protocol. Their characteristics are listed in Table 1. Patient ages were 51–80, with a median of 71 years old. They had 14 vaginal tumors, 6 vulval tumors and 3 cervical uteri tumors. Four of the patients' tumors were recurrence after surgery, and two of them received adjuvant chemotherapy. The GTV ranged from 0.27 to 211.1 cm^3^, with a mean of 34.2 cm^3^ and a median of 5.8 cm^3^. The median GTV volumes were 63 cm^3^ in the uterine cervix, 14 cm^3^ in the vagina and 0.4 cm^3^ in the vulva.

**Table 1.** Patient and tumor characteristics

No. of cases		23	(100%)
Age (median)		51–80 y (71 y)	
Performance status	0	20	(87.0)
	1	2	(8.7)
	2	1	(4.3)
Tumor site	Vagina	14	(60.9)
	Vulva	6	(17.4)
	Cervix Uteri	3	(13.0)
UICC T stage	T1	6	(26.1)
(include rT)	T2	13	(56.5)
	T3	4	(17.4)
Metastasis	Lymph node	5	(21.7)
	Distant	1	(4.3)
Pervious treatment	Surgery	4	(17.4)
	Systemic therapy	3	(13.0)

According to the UICC classification of each gynecological site including rTN, there were 5 T1N0, one T1N1, 11 T2N0, 2 T2N1, 2 T3N0 and 2 T3N1. There was one rT1 and two rT2 cases, previously treated with surgery. One T1 patient received partial resection and three patients received chemotherapy before referral to our hospital. One initial case of T3N1 disease had a solitary 3 mm lung metastasis. According to the UICC classification of skin melanoma including rTN, there were 4 T3aN0, 13 T4aN0, 5 T4aN1 and 1 T4aN2 cases, and one of the T4aN1 patients had M1. The follow-up period of all patients ranged from 6–53 months, with a median of 17 months. The median follow-up period for surviving patients is 24 months.

### Treatment characteristics

Of the 23 patients, 22 (96%) were irradiated with a total dose of 57.6 GyE and one (4%) was irradiated with 64.0 GyE. The overall treatment time for the 23 patients ranged from 24–28 d, with a median of 25 d.

**Fig. 1.**
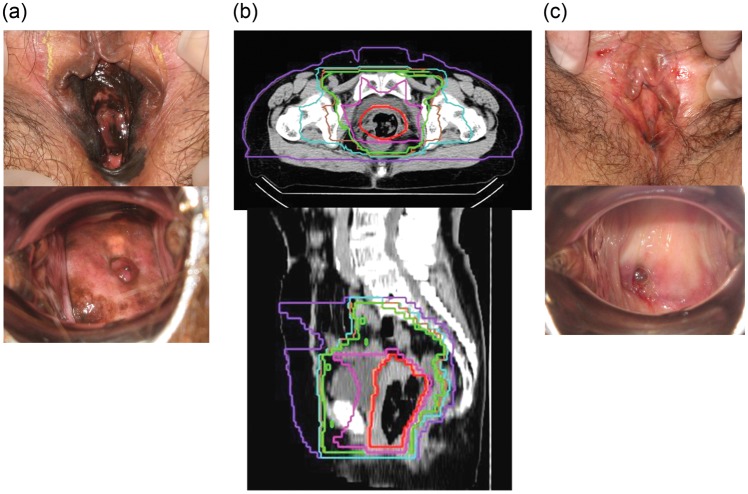
A case of T3N0M0 vulval MM treated with C-ion RT. (**a**) Genital examination findings before treatment. (**b**) Radiation field and dose distribution on axial and sagittal CT images. (**c**) One year after the treatment. The patient had irradiated 36 GyE to small pelvis and inguinal region, then reduced irradiation field to the tumor site up to a total of 67.6 GyE. Isodose curves are 96% (red), 90% (orange), 70% (pink), 60% (yellow green), 50% (green), 30% (cyan) and 10% (purple) of 57.6 GyE. She has been living without recurrence or adverse effect for 2 years.

The CTV1 ranged from 287.1–1482.1 cm^3^, with a mean of 1141.6 cm^3^ and a median of 1142.4 cm^3^. The CTV2 ranged from 51.6–509.6 cm^3^, with a mean of 216.2 cm^3^ and a median of 175.4 cm^3^. Figure 1 shows the typical radiation field with dose distribution. Of the 23 patients, 22 were irradiated in three-portal passive beams and 1 patient was irradiated in two-dimension scanning beams. Figure 1 shows the typical radiation fields and genital examination photos of the tumors pre and post treatment.

One patient received DAV just before C-ion RT, and she received concurrent Feron during C-ion RT in our hospital at the request of the referring gynecologist.

Another patient (the first case) received concurrent DAV, although the other 21 patients underwent no concurrent therapy. With regard to adjuvant therapies, five patients received DAV, three received DAVFeron and two received Feron.

### Toxicity

No acute reactions of Grade 3 or worse were observed, except in one patient (4%) (Table 2). In that patient, Grade 3 acute reactions of urinary tract pain with bladder bleeding and Grade 3 late reactions of bowel, bladder and skin were observed after treatment with induction DAV and concurrent Feron. Her Grade 3 late skin reaction (ulcer) occurred after inguinal lymph node biopsy, 15 months after the completion of C-ion RT. Grade 2 and Grade 1 late bowel reactions were observed in two parents and one patient, respectively. A Grade 1 late bladder reaction was observed in one patient, and a Grade 1 late edema was observed in four patients.

**Table 2.** Acute adverse effects

Grade	0	1	2	3	4
Colitis	8	11	4	0	0
Diarrhea	8	11	4	0	0
Fecal incontinence	22	3	1	0	0
Malaise	14	8	1	0	0
Dermatitis	2	10	10	0	0
Urinary frequency	11	11	1	0	0
Urinary infection	22	1	0	0	0
Urinary tract pain	4	10	8	1	0
Perineal pain	2	11	10	0	0
Vaginal pain	3	11	9	0	0

CTCAE version 4.0.

### Tumor control

In terms of initial tumor response, maximum reaction within 6 months after the start of C-ion RT, was complete response (CR) in 6 and partial response (PR) in 17. One M1 (solitary lung) patient treated with concurrent DAV achieved CR. Six patients had local recurrence, with 3 in CTV and 3 in marginal region. Five patients had recurrence in regional lymph nodes and 11 patients in distant organs. The patient with Grade 3 acute and late reactions had no local or regional recurrence, but had lung metastases 15 months after the completion of C-ion RT.

**Fig. 2.**
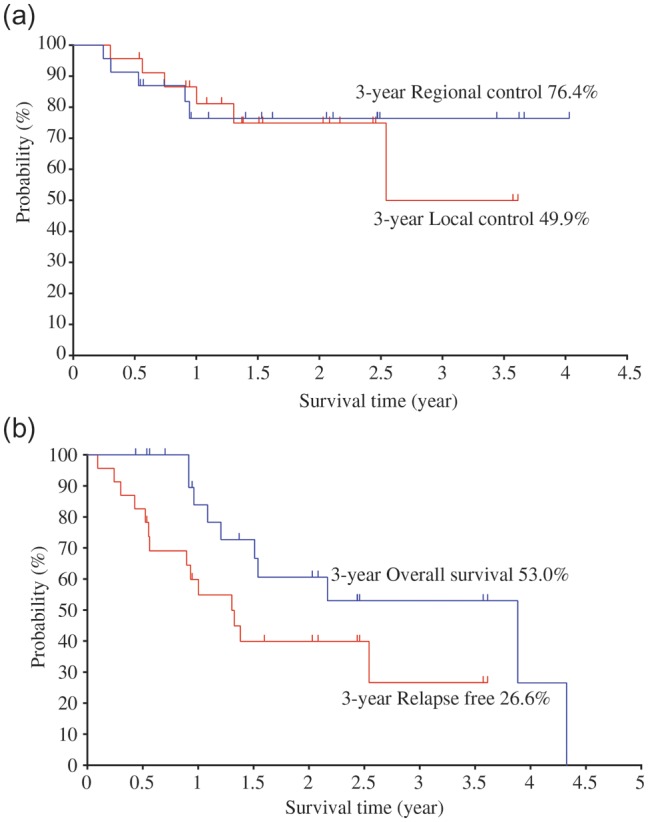
(**a**) Local control (LC) and regional control (RC) rates, (**b**) relapse-free survival (RF) and overall survival (OS) rates in all patients treated with C-ion RT.

Table 3 shows the 3-year LC, RC and DC rates for various categories. The 3-year LC, RC and DC rates of the 23 patients were 49.9, 76.1 and 40.1%, respectively. There were statistical differences in LC between the uterine cervix (0% at 1.3 years) and the vagina (55.8% at 3 years) (*P* = 0.009), and in LC between uterine cervix and the vulva (100% at 2.5 years) (*P* = 0.027). However, there was no significant difference in LC, RC or DC associated with UICC T N classification, tumor volume ≥60ml or <60ml, or with or without systemic therapy. Figure 2a shows the LC and RC curves for all patients.

**Table 3.** Three-year local control (LC), regional control (RC), and distant-free (DF), relapse-free (RF) and overall survival (OS) rates (%) by various factors

Group (No. of cases)	LC	RC	DF	FR	OS
All (23)	49.9	76.1	40.1	26.6	53.0
Uterus (3)	0	66.7	66.7	0	50.0 (2.4 y)
Vagina (14)	55.8	77.1	36.2	26.5	62.3
Vulva (6)	100	80.0	62.5	66.7	30.0 (2.5 y)
T1 (6)	75.0	80.0	27.8	31.2	50.0
T2 (13)	33.7	67.3	53.6	19.2	70.0
T3 (4)	100	100	50.0	50.0	50.0 (2.2 y)
Melanoma T3a (4)	100	100	75.0	75.0	66.7
Melanoma T4a (19)	35.1	71.3	33.1	17.0	50.3
N0 (18)	47.3	81.6	50.9	31.0	67.3
N positive (5)	80.0 (2.1 y)	60.0 (1.6 y)	20.0 (1.6 y)	20.0 (1.6 y)	33.3 (2.1 y)
≥60 cm^3^ (17)	53.7	68.6	44.1	32.9	59.0
<60 cm^3^ (6)	55.6	100	33.3	16.7	40.0 (2.4 y)
Systemic therapy (13)	36.4	83.1	32.1	17.3	55.0
No systemic (10)	78.8	66.7	63.0	46.7	47.6 (2.4 y)

*P = 0.009, **P = 0.027.

### Relapse free and overall survival

In summary, 14 patients had recurrence at some sites, 8 patients died from MM, and 5 patients are still living with MM. Two patients died of intercurrent disease, one with and one without MM recurrence, so a total of 10 patients died. The 3-year RF rate for the 23 patients was 26.6% and the OS was 53.0%. RF and OS curves are shown in Fig. 2b. Table 3 shows the 3-year RF and OS rates for various categories. There was no significant difference in RF or OS associated with UICC T N classification, tumor site, tumor volume ≥60ml or <60ml, or with or without systemic therapy.

## DISCUSSION

In this report, the patient median age of 71 years old was higher than in other reports [15–22]. The standard treatment for MM of the gynecological region has until recently been radical surgery with regional lymphoadenectomy, depending on lesion size, thickness, and depth of invasion [2–4, 15–22]. However, many studies have concluded that radical surgery can be associated with physical disabilities and does not improve the survival rate of patients with advanced tumors. Patients with lesions deeper than 4 mm have a high risk of distant metastases, even with radical surgery [17, 18]. We normally suggest that elderly patients and their primary attending physicians take a conservative treatment approach in the light of this fact.

The results are less favorable for LC and OS for malignancies of the cervix uteri compared with the vagina and the vulva due to large tumor volumes that may extend to a considerable depth. The median GTV volume in uterine cervix (63 cm^3^) could not be controlled, even if irradiated with 64 GyE. However, tumor control was not significantly different for tumor volume ≥60 ml or <60 ml, and it is thought that other factors influenced the prognosis of MM of the vulva and the vagina. In gynecological MM, the outcome may be affected by the tumor site and spread as well as volume. Factors affecting prognosis were not identified in this number of cases with a range of backgrounds. Systemic therapy had no influence on the treatment outcome in this series.

Table 4 lists reviews of the literature, in which patients were mainly treated with surgery. Five-year survivals are reported as 20–91%, dependent on various factors, mainly tumor volume, extension and primary site. MM is considered a radioresistant tumor, so we could not find any literature about treatment of gynecological MM with conventional photon radiotherapy. We think the 53% 3-year survival rate we obtained using C-ion RT indicates that it is worthy of consideration as a conservative treatment option.

**Table 4.** Review of literature

Institute	Reported year	No.	Site	Mean age	Treatment modality	Survival rate	
MD Anderson (13)	2010	37	vagina	60.6	surgery chemotherapy radiotherapy	5-year overall	20%
MD Anderson (14)	2001	51	vulva	54	mainly surgery	5-year Stage I 5-year Stage II − IV	91% 31%
National Cancer Data Base (15)	1999	569	vulva	66	mainly surgery	5-year relative^a^	62%
Swedish National Cancer Registry (16)	1999	219	vulva		mainly surgery	5-year relative^a^	47%
Duke University (17)	1998	16	vulva 30 vagina 9 cervix 4	66	mainly surgery	3-year overall 5-year overall	71% 54%
Indian University (18)	1993	16	vulva	59	mainly surgery	5-year overall	30%
Memorial Sloan-Kettering (19)	1992	80	vulva		surgery	10-year by thickness < 0.75 mm 0.75 − 1.5 mm 1.51 − 3.0 mm > 3.0 mm	48% 68% 44% 22%
Birmingham (20)	1990	16	vulva	50		5-year overall	35%
NIRS (Present study)	2013	23	vulva 6 vagina 14 cervix 3	71	carbon ion radiotherapy	3-year overall	53%

^a^The author said that relative survival rates were used because the computation procedure adjusts the cumulative observed actual survival rates.

Comparing the LC achieved here with our institute experience of C-ion RT for choroidal melanoma and head and neck MM, gynecological melanoma has a poor outcome. The 5-year LC and OS were 92.8 and 80.4% in advanced or unfavorably located choroidal melanoma and 75 and 35% in MM of head and neck, respectively [7, 8]. The results of C-ion RT treatment of gynecological melanoma were 49.9% for 3-year LC and 53.0% for OS. We suggest that deep tumor extension may affect the prognosis of gynecological MM. Further treatment strategies would be necessary to improve the treatment outcome. For example, more precise diagnosis of tumor extension, dose escalation with a more conformal beam, more precise radiation field setting for local improvement, and combination with more effective systemic therapy to suppress systemic metastasis may be considered.

In regard to acute and late toxicities, one patient developed Grade 3 reactions in the bowel, urinary tract, and skin. Although she received induction DAV and concurrent Feron, our experience with C-ion RT to head and neck MM with DAV had previously revealed no such severe adverse effects. We cannot prove the concurrent Feron was the cause of the Grade 3 reactions, although the results indicate there may be a relationship.

Until now, our efforts have been directed toward increasing of the patient in order to produce results that can provide cogent clinical evidence. Although the median follow-up periods of 17 months (all patients) and 24 (surviving patients) are still short, we believe this article is the first case series to describe the results of C-ion RT for gynecological melanoma. Careful investigation and observation is necessary because Grade-3 bowel, urinary tract and skin adverse effects were observed in one patient with indeterminate cause.

## CONCLUSION

In a clinical trial of C-ion RT for gynecological melanoma, we observed a similar therapeutic effectiveness to that of surgery, with an acceptable rate of morbidity of normal tissues. C-ion RT could become a non-invasive alternative to radical treatment for this intractable tumor.

## CONFLICT OF INTEREST

The authors declare that there are no conflicts of interest.
